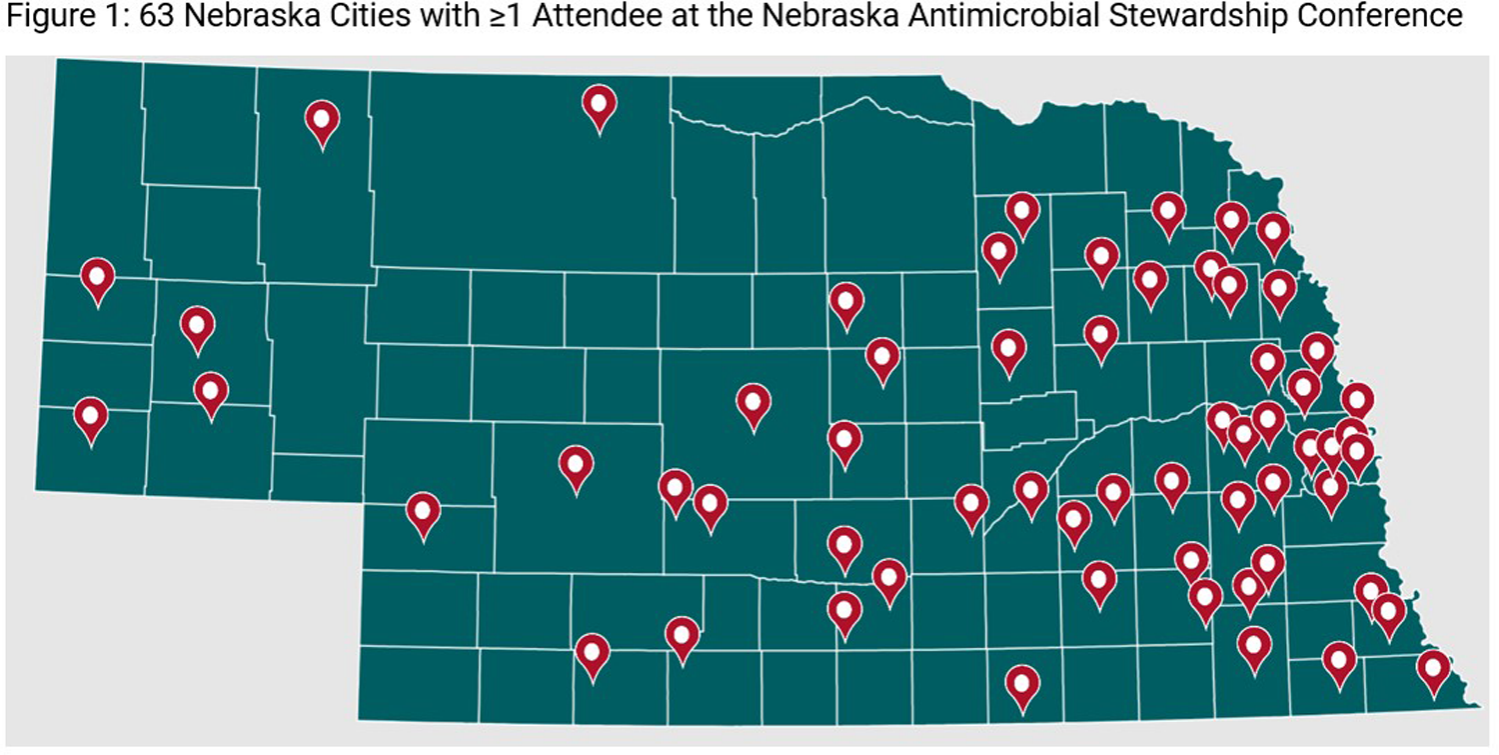# Antimicrobial Stewardship Practice Changes Following a Statewide Educational Conference in Nebraska

**DOI:** 10.1017/ash.2024.147

**Published:** 2024-09-16

**Authors:** Jenna Preusker, Mounica Soma, Scott Bergman, Danny Schroeder, Kate Tyner, Trevor Van Schooneveld, Matthew Donahue, Muhammad Salman Ashraf, Juan Teran Plasencia

**Affiliations:** Nebraska Medicine; University of Nebraska Medical Center; Nebraska ASAP; The Nebraska Medical Center; Nebraska Department of Health and Human Services

## Abstract

**Background:** In 2023, Nebraska held its 4th state antimicrobial stewardship (AS) educational conference, an annual one-day in-person event with continuing education offered for nurses, pharmacists, microbiology lab technicians, and physicians. One challenge of educational events is determining if content has been translated into practice. We sought to assess AS-related practice changes implemented by conference attendees. **Methods:** Conference attendees were sent 2 surveys by email following the conference. Survey 1 questions were integrated into the continuing education credit evaluation immediately following the conference. Survey 2 was sent three months later to all registered attendees. Qualitative responses were grouped by theme and descriptive statistics were used to evaluate **Results:** There were 203 attendees from across the state including a diverse group of learners (Table 1) representing metropolitan and rural areas of Nebraska (Figure 1) from acute care hospitals, critical access hospitals, long-term care settings, and public health. A total of 148 attendees (73%) answered questions in Survey 1 (Table 2), and 79 (39%) attendees responded to Survey 2. On Survey 1, 94% of respondents indicated that they intended to make practice changes, though 60% anticipated barriers including further staff training needs and lack of resources and health system support. On Survey 2, 83% of respondents indicated successful implementation of practice changes at three months after the conference. The most common practice changes included enhanced communication strategies, improved antibiotic tracking, monitoring, and review, policy and procedure updates, and AS tool implementation. On Survey 1, 26% (35/131) strongly agreed that their ability to treat patients was adequate prior to the conference; this increased to 55% (72/131) post-conference. On Survey 2, 56% (22/39) of respondents reported improvement in patient outcomes because of implemented practice changes following conference attendance. However, some also mentioned a short follow-up survey timeline as a limitation in assessing patient outcome improvements. Reported outcomes included improved receptiveness from providers, patients, and families to antibiotic use recommendations, shorter prescribed durations, and more appropriate initial antibiotic selection. Improved team performance was noted by 73% (27/37) of respondents. Themes included improved communication with internal and external stakeholders, more collaborative team discussions, increased confidence in recommendations, expanded provider and staff engagement, and increased leadership involvement. **Conclusions:** In addition to improved knowledge and understanding for a variety of AS-related areas, attendees of the conference also reported a high rate of practice changes that led to perceived improvements in patient outcomes and team function.

**Figure:**
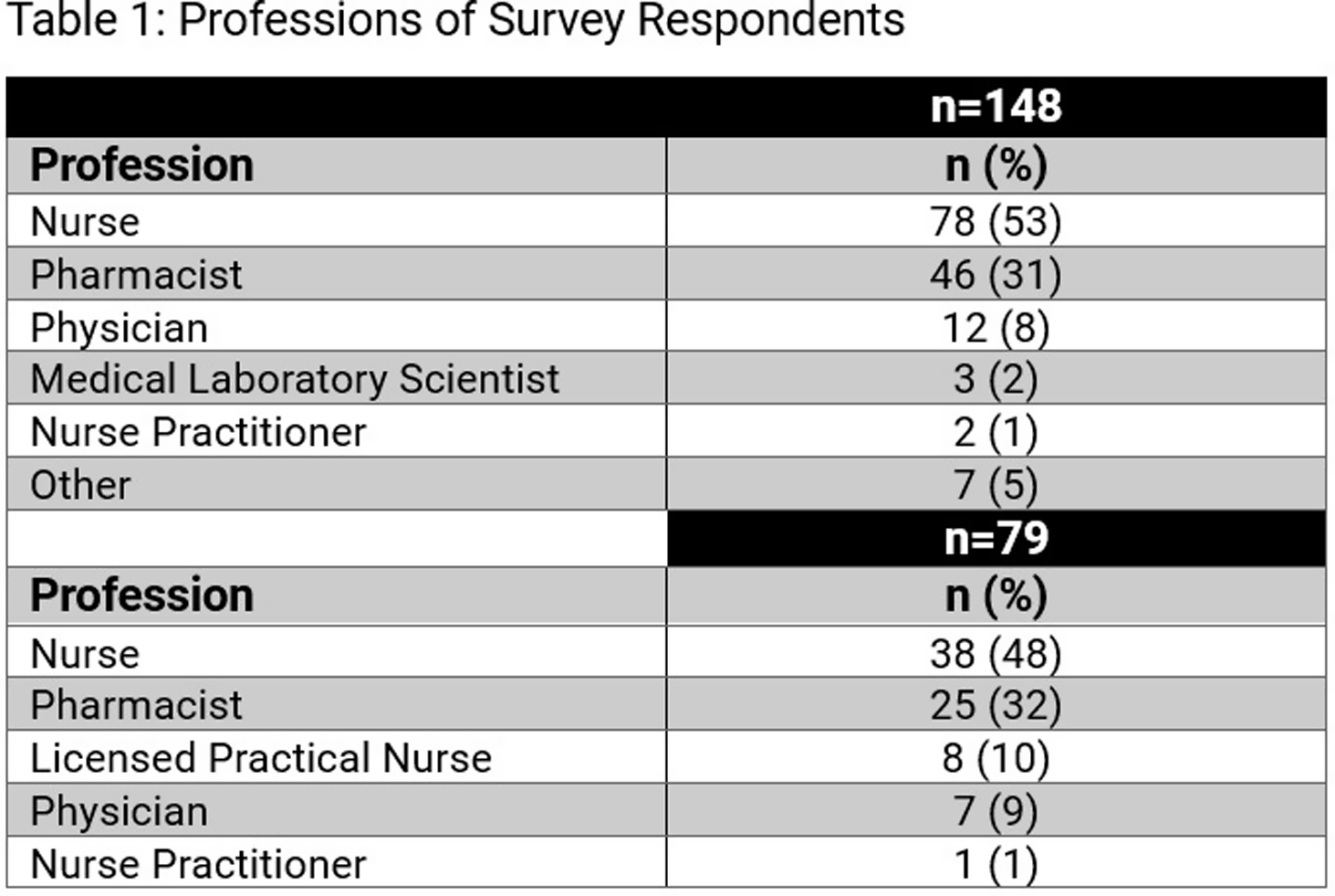

**Figure:**
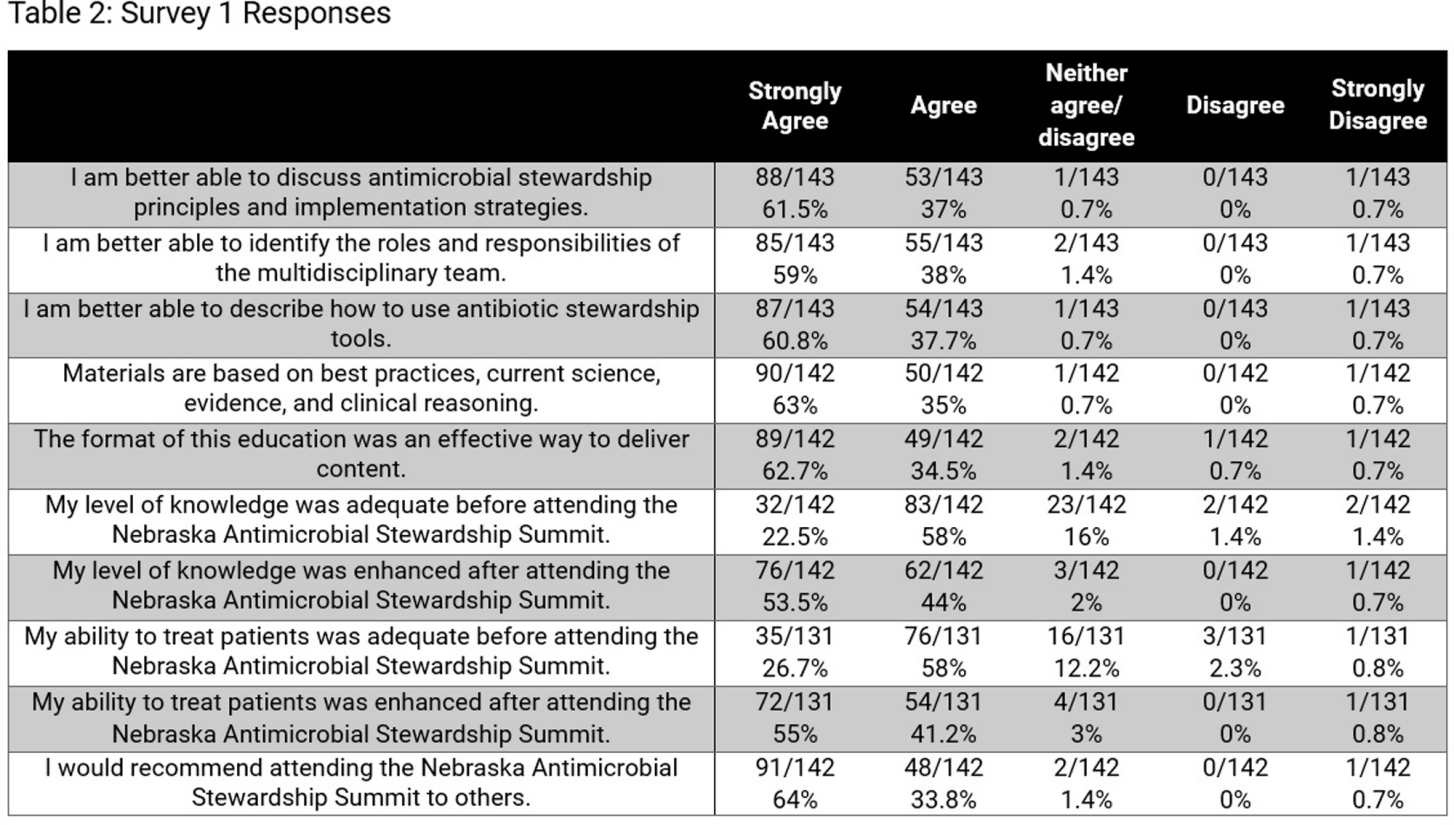

**Figure:**